# A nutritional supplement based on a synbiotic combination of *Bacillus subtilis* DSM 32315 and L-alanyl-L-glutamine improves glucose metabolism in healthy prediabetic subjects – A real-life post-marketing study

**DOI:** 10.3389/fnut.2022.1001419

**Published:** 2022-12-08

**Authors:** Anna Kordowski, Vivian Valeska Tetzlaff-Lelleck, Bodo Speckmann, Gunnar Loh, Axel Künstner, Franziska Schulz, Torsten Schröder, Martin Smollich, Christian Sina, Heike tom Dieck

**Affiliations:** ^1^Institute of Nutritional Medicine, University Hospital Schleswig-Holstein, University of Lübeck, Lübeck, Germany; ^2^Evonik Operations GmbH, Hanau-Wolfgang, Germany; ^3^Perfood GmbH, Research and Development, Lübeck, Germany

**Keywords:** probiotic, synbiotic, colon-targeted delivery, microbiome modulation, impaired glucose tolerance, prediabetes

## Abstract

**Introduction:**

Impaired glucose homeostasis is a significant risk factor for cardiometabolic diseases, whereas the efficacy of available standard therapies is limited, mainly because of poor adherence. This post-marketing study assessed the glucose-lowering potential of a synbiotic-based formulation.

**Methods:**

One hundred ninety-two participants were enrolled in a digital nutrition program with continuous glucose monitoring (CGM) and received a study product comprising *Bacillus subtilis* DSM 32315 and L-alanyl-L-glutamine. Participants underwent a first sensor phase without supplementation, followed by a 14-day supplementation phase without sensor, and completed by a second sensor phase while continuing supplementation. Fasting glucose levels were determined before and after supplementation by CGM. In addition, the postprandial glycemic response to an oral glucose challenge, body weight, HbA1c concentrations, and BMI was analyzed. Subgroup analyses of subjects with elevated glucose and HbA1c levels vs. normoglycemic subjects were performed.

**Results:**

Supplementation with the study product resulted in significant improvements in glucose parameters (delta values: fasting glucose –2,13% ± 8.86; iAUC_0–120_ –4.91% ± 78.87; HbA1c: –1.20% ± 4.72) accompanied by a significant weight reduction (−1.07 kg ± 2.30) in the study population. Subgroup analyses revealed that the improvements were mainly attributed to a prediabetic subgroup with elevated fasting glucose and HbA1c values before supplementation (delta values: fasting glucose −6.10% 4± 7.89; iAUC_0–120_ –6.28% ± 115.85; HbA1c −3.31% ± 4.36; weight: −1.47 kg ± 2.82).

**Conclusion:**

This study indicates that the synbiotic composition is an effective and convenient approach to counteract hyperglycemia. Further placebo-controlled studies are warranted to test its efficacy in the treatment of cardiometabolic diseases.

## Introduction

Optimal maintenance of blood glucose homeostasis is essential for the prevention of hyperglycemia. Different metabolic parameters such as impaired fasting glucose (IFG) concentrations or elevated levels of glycated hemoglobin A1c (HbA1c) can serve as predictors of impaired glucose homeostasis. According to the American Diabetes Association (ADA), hyperglycemia is defined by fasting blood glucose concentrations of 100 to 125 mg/dL and an HbA1c level of 5.7 to 6.4%. This intermediate state of hyperglycemia is a risk factor for the development of cardiometabolic diseases such as type 2 diabetes mellitus (T2D) (1–4). As the prevalence of hyperglycemia increases in the general population (5), so does the related economic burden on healthcare systems. Therefore, there is an urgent need for effective prevention strategies (6–8). As dietary factors directly affect blood glucose levels, the modification of the individual diet is the main target in hyperglycemia management (9, 10). Strategies such as calorie restriction and low-glycemic diets – even though being very effective in lowering postprandial blood glucose levels (11, 12) – require high compliance by hyperglycemic patients. It has been shown that the respective dietary interventions cause consistent changes in gut microbiota (13). The intestinal microbiota and its metabolic products such as butyrate also play a crucial role in the individual metabolic response to food. Accordingly, complex interactions between the gut microbiota and blood glucose homeostasis have been proven, suggesting that the specific microbiota modulation might represent an innovative and suitable approach to improve blood glucose regulation (13–15).

An established method to achieve favorable changes in the microbiota and stimulate butyrate formation is the intake of prebiotic fibers, either through a regular diet or fortified foods or supplements (16). In this context, the most studied prebiotics belong to the group of fermentable oligosaccharides, disaccharides, monosaccharides, and polyols (FODMAPs). However, to yield respective effects, these carbohydrates must be consumed in high amounts and often cause adverse effects such as diarrhea, constipation, and flatulence (17, 18). Therefore, low-FODMAP diets have become popular as they effectively reduce symptoms in patients with irritable bowel syndrome (IBS), thus limiting the use of dietary fibers as a source for intestinal butyrate production, at least for specific populations (19, 20). An alternative approach is the direct application of butyrate-producing bacteria. Recently, a synbiotic-based formulation containing *Bacillus subtilis* DSM 32315 and L-alanyl-L-glutamine (Ala-Gln) has been developed to shift the composition and activity of the gut microbiome toward butyrate production (21). The butyrogenic activity of Ala-Gln can be attributed to glutamine which is hydrolyzed to glutamic acid and then catabolized to butyric acid. The alanine residue increases the stability of the dipeptide, serves as a spore germination trigger, and supports *Bacillus subtilis* metabolism (21, 22). Compared to known butyrate producing taxa, *Bacillus* is not strictly anaerobic, but aerotolerant/microaerophilic, which makes it more robust both during technological processing and gastrointestinal passage. Although *Bacillus* species are not direct butyrate producers, some can indirectly trigger microbial butyrate production in the gut (e.g., *Bacillus subtilis*, *Bacillus coagulans* and *Bacillus licheniformis*) (23, 24). A pilot study showed that this synbiotic-based formulation resulted in significantly increased levels of butyrate in stool and butyrate-producing taxa. In addition, circulating lipid parameters (LDL, total cholesterol, LDL/HDL ratio) were significantly reduced and further metabolic effects such as glucose modulation were observed after the application of the synbiotic (21). Consequently, it was of major medical and scientific interest to investigate the metabolic effects of this formulation in a larger cohort. Since it can be expected that the metabolic and microbiome-modulating responses to the synbiotic-based formulation significantly vary interindividual, a real-world study design was chosen. This setting has the important advantage that – unlike in controlled clinical settings – the impact of individual lifestyle habits can be taken into consideration (25).

Therefore, in this retrospective analysis, we investigated whether an intervention with the synbiotic-based product might be an effective strategy to improve glycemic control and alter the gut microbiota in a real-world setting. In addition, we assessed differential individual responses and sought to identify subgroups that would benefit most from the intervention.

## Materials and methods

### Intention of the investigation

The main objective of this observational study was to investigate the influence of a synbiotic-based dietary supplement (SAMANA^®^ FORCE, Evonik Operations GmbH, Darmstadt, Germany) on metabolic parameters (fasting glucose level, post-prandial glycemic response to glucose, test meals and everyday meals, average glucose level, blood sugar variability, calculated HbA1c concentrations). Additionally, the effects of supplementation on gut microbiota, body weight, BMI, waist-to-hip ratio (WHR), as well as tolerability and stool characteristics have been investigated. Subjective effects on non-specific gut symptoms, appetite and cravings, performance, concentration, general well-being and intake behavior have been assessed as well.

The Institute of Nutritional Medicine at the University of Lübeck was asked to evaluate the data regarding human metabolism in order to subsequently prepare prospective clinical trials. The ethics committee of the University of Lübeck approved the retrospective analysis of the data presented in this paper (AZ 20-415 “Analysis of postprandial tissue glucose responses to different foods”).

### Study product

The study product consisted of HPMC capsules comprising 2 × 10^9^ CFU (colony forming unit) *Bacillus subtilis* DSM 32315 (Evonik Industries AG, Essen, Germany), 290 mg L-alanyl-L-glutamine, 90 mg Curcuma extract (approx. 70–80% curcumin), 90 mg green tea extract (approx. 50% EGCG), 5 mg zinc, 0.56 mg vitamin B6, 20 μg D-biotin, 0.75 μg vitamin B12, 4 μg vitamin D, 2.4 mg pantothenic acid (=content per recommended daily dose of two capsules, size 1). Of note, vitamin B12 serves as a cofactor of the enzyme glutamate mutase, which catalyzes the first step of glutamate degradation toward pyruvate, and it was therefore added to stimulate microbial butyrate formation further. Capsules were coated with a methyl methacrylate-based polymer (EUDRAGUARD^®^ biotic; E1207, Evonik Operations GmbH, Darmstadt, Germany) with pH-dependent solubility to enable a colon-specific delivery of the capsule content. This allows the capsule to be taken independently of food, with or without a meal, and both together at the same time or at different times during the day. According to German legal requirements, the study product has been notified as a dietary supplement.

### Study design

The study was conducted as a real-life, open-label, exploratory human study (post-marketing) between July and December 2021 by Perfood GmbH (Lübeck, Germany) with retrospective data analysis. Perfood GmbH is a company offering an app-based digital nutrition program (MillionFriends) with continuous glucose monitoring (CGM). The study was designed as a single-arm study, meaning that all the participants received the same treatment and there was no control group. All participants gave their informed consent to their anonymized data being used for scientific purposes.

In total, 192 volunteers from the general population were recruited using different social media channels (Facebook, MillionFriends newsletter). As part of a digital nutrition program,^1^ participants took part in a 14-day test phase in which they recorded their meals and physical activity via an app (MillionFriends App, Perfood GmbH) and consumed defined standardized test meals. During this period, the participants wore a glucose sensor for CGM. After the first sensor-assisted phase, the participants started the intervention with the supplement (two unchewed capsules/day). The questionnaire about the intake behavior and adherence was filled in on a weekly basis. The participants were considered adherent if they took at least ten capsules per week for three out of four weeks. Apart from that, the participants continued their diet as usual. After two weeks of supplementation, the second 14-days sensor-assisted test phase started. The intake of the synbiotic-containing supplement continued during this sensor phase. Glycemic responses to the test meals and parameters describing blood glucose were measured again. Furthermore, at the beginning of each sensor phase a standardized glucose tolerance test was performed.

**FIGURE 1:**
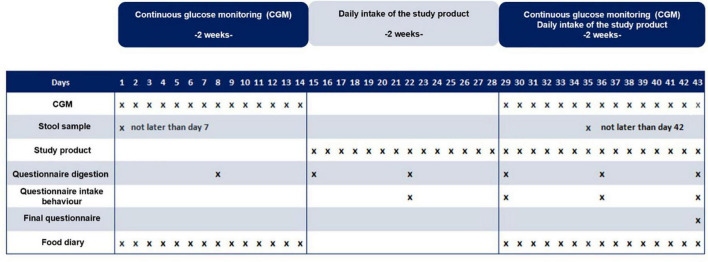
Study design flow chart. The study was designed as a single-arm study.

During the whole intervention, participants also answered questions on their digestion and general well-being. In addition, participants were asked to take stool samples at the beginning and at the end of the test phase and to send them in for analysis. The study design and flow chart are depicted in Figure 1.

### Glucose measurement

CGM was performed for 14 days during the test phase using the Abbott FreeStyle Libre system (Abbott GmbH, Wiesbaden, Germany), including one day for sensor calibration. Baseline values of the parameters of interest were measured within these 14 days. This included the participants’ blood glucose responses to prescribed and standardized test meals and other parameters describing blood glucose. Fasting blood glucose was determined using a specifically developed algorithm as the baseline blood glucose value. The baseline value is predicted using the daily individual 24-h glucose profiles and the medical history data. The HbA1c value was calculated by multiplying the average glucose level of the entire test phase by 0.03 and adding 2.60 (26). It should be noted that the HbA1c value calculated in this study is not of the same quality as the HbA1c value measured in standardized laboratories. To analyze the glycemic responses after glucose intake, the iAUC (incremental area under the curve) was determined 120 min after the logged food intake by applying the trapezoidal rule. Participants were instructed to maintain a minimum interval of 2 h between two meals and/or a meal and physical activity.

To determine glycemic responses to a standardized glucose test meal the participants were asked to perform a standardized glucose test in both sensor-assisted test phases. For this, they received two 60 g bags of glucose with their test kit. Participants were instructed to dissolve the glucose powder in 200–300 ml water and drink it in the morning on an empty stomach at the beginning of each sensor phase. The compliance was determined by means of an app questionnaire.

### Fecal microbiota analysis

The composition of the gut microbiome was analyzed using 16S ribosomal DNA sequencing. Therefore, participants provided a random sample from a bowel movement in the first and last week of the intervention. The samples were stored in a PSP Spin Stool DNA Plus kit provided by Invitek Molecular GmbH (Berlin, Germany) with an 8 ml DNA stabilizer. After arrival, samples were stored at –80°C. Extraction, isolation, and sequencing of the V3V4 region of the 16S rRNA gene were performed at Microsynth AG (Balgach, Switzerland). After sequencing, the microbiome data were preprocessed with dada2 (v1.20.0). Covariates were obtained from Perfood GmbH. The raw data were trimmed to 220 bp and quality filtered (maxEE = 1). Reads were merged, and chimeras were removed (proportion of non-chimeric sequences 0.9918). The taxonomic assignment was done using RDP v18 as a reference database (IdTaxa, DECIPHER v2.20.0). Preprocessed files were analyzed using R (v4.1.2). Alpha diversity was estimated using the Shannon index (vegan v2.5-7). Furthermore, Pielou’s evenness and observed richness were estimated using rarefied data (3,742 contigs) using the R packages microbiome (v1.18.0) and phyloseq (v1.40.0), respectively. Beta diversity was calculated using Euclidean distance on centered log-ratio (*clr*) transformed counts (i.e., Aitchison distance). Differential abundance analysis was performed using corncob (v0.2.0; fdr < 0.05) on counts, while correlation analysis was performed using scaled counts (proportions) and Spearman’s rho. Amplicon sequencing data used for this study were submitted to the European Nucleotide Archive (ENA) and are available under accession number PRJEB55518.

### Questionnaires

The participants were asked to fill out questionnaires on a regular basis (Figure 1). Of interest was whether taking the supplement had an effect on the participants’ digestion. To monitor the changes, they filled out a questionnaire about their digestion every week. The questionnaire included questions about the frequency and consistency of bowel movements. In addition, questions were asked about the presence of various digestive complaints such as abdominal pain, flatulence or diarrhea, as well as their frequency and the burden associated with them. Finally, the feeling of hunger and satiety was also recorded. In addition, the expression of various lifestyle parameters such as well-being, concentration and physical performance was measured weekly on a scale from 0 (very poor) to 10 (very good). At the beginning of the consumption of the capsules, the participants answered weekly questions about their intake behavior. This was to check whether the participants were consuming the investigated product regularly. A final questionnaire had to be filled out at the end of the study. This questionnaire asked about the tolerability of the product and possible positive effects that could be observed in the course of taking the product.

### Statistical analysis

Statistical analyses were performed using R (v3.6.2). For comparison of the glucose levels, paired t-test was used unless the normal distribution could not be assumed. In such a case, non-parametric Wilcoxon test was used for the analysis. To test for the normal distribution, the Shapiro-Wilk test was applied. Statistical significances were reported using uncorrected p-values or p-values that were corrected for multiple testing using Benjamini–Hochberg correction (denoted as q-values). Differences in beta diversity were assessed by permutational multivariate analysis of variance (PERMANOVA) using distance matrices with 9,999 permutations to calculate significance values. Delta (%) presented in the tables is a mean of the percentage change calculated for each participant for the respective parameter.

## Results

### Cohort description

Via social media channels (Facebook, MillionFriends newsletter) 192 study participants were recruited. The study cohort consisted of 65.10% women (*n* = 125) and 34.90% men (*n* = 67). The mean age was 42.80 years (±12.48). The mean weight was 80.28 kg (±17.88) with a mean BMI of 26.88 kg/m^2^ (±6.17). The demographic data of the participants are presented in Table 1. To exclude changes in eating behavior as a confounding factor, the occurrence of significant differences in eating behavior in the first and second sensor phase was analyzed based on the documentation. Neither energy nor the average daily intake of protein and fat per day differed significantly between the two sensor phases. However, the average daily intake of carbohydrates (*p* = 0.008) and fiber (*p* = 0.002) per day was significantly lower in the second sensor phase. Similarly, the average proportion of carbohydrates in the average energy intake decreased (*p* < 0.001), while it increased for fats (*p* < 0.001, Supplementary Table 1). These differences, though statistically significant are very low and in the range of normal daily fluctuations. They could be explained by the fact that the participants could observe changes in their glucose level after each meal in the app provided with the sensor and might have tried to adjust their diet on their own. Regarding physical activity, participants were less active during the second sensor phase (*p* < 0.001). This effect could be related to the motivation of the participants in logging their physical activity, as it is not recorded automatically.

**TABLE 1:** Study cohort.

Variable	Cohort (*n* = 192)
**Gender**	
Female (%)	125 (65.10%)
Male (%)	67 (34.90%)
Age (SD)	42.80 (±12.46)
Weight in kg (SD)	80.28 (±17.88)
BMI kg/m^2^ (SD)	26.88 (±6.17)
**Weight classification**	
Underweight (%)	5 (2.60%)
Normal weight (%)	87 (45.31%)
Overweight (%)	54 (28.13%)
Obesity WHO I (%)	25 (13.02%)
Obesity WHO II (%)	14 (7.29%)
Obesity WHO III (%)	7 (3.65%)

Baseline characteristics of the test cohort. SD – standard deviation.

### Subgroup analysis

For the subgroup analyses, only patients with glucose data available for both measurement time points (from both sensor phases) were included (*n* = 180). In 99 participants, the calculated HbA1c value was above 5.7%, while the fasting blood glucose was above 100 mg/dL in 62 participants. Since a fasting blood glucose above 100 mg/dL and HbA1c values above 5.7% indicate the presence of prediabetes, the participants could be divided into a prediabetic (*n* = 62) and a non-prediabetic (*n* = 118) subgroup. Prediabetic participants were 53.23% female (*n* = 33) and 46.03% male (*n* = 29). The mean age was 42.52 (±12.18) years. The mean weight was 84.18 kg (±16.20) with a mean BMI of 27.60 kg/m^2^ (±6.45). Non-prediabetic participants were in 69.49% female (*n* = 82) and 30.51% male (*n* = 36). The mean age was 41.16 (±12.10) years. The mean weight was 78.13 kg (±18.35) with a mean BMI of 26.45 kg/m^2^ (±5.98). These two groups were evaluated separately (Supplementary Table 2).

Neither the energy nor the average amount of carbohydrates, protein, fat, and fiber per day differed significantly between the two sensor phases in prediabetic patients. However, the average proportion of carbohydrates in the daily calorie intake decreased (*p* = 0.025), while the average proportion of fats increased (*p* = 0.035, Supplementary Table 3). Regarding the intake of macronutrients of non-prediabetic participants, no significant difference was found regarding the average daily intake of fat, protein, or energy. However, the intake of carbohydrates (*p* = 0.001) and fibers (*p* < 0.001) was reduced in the second sensor phase. Additionally, the average percentage of carbohydrates in the average daily calorie intake decreased (*p* < 0.001), whereas the average percentage of fats increased (*p* < 0.001). Regarding physical activity, participants with prediabetes (*p* = 0.016) and without prediabetes (*p* < 0.001, Supplementary Table 4) were less active during the second sensor phase.

### Adherence to capsule intake

The participants were instructed to take two capsules per day over four weeks. According to the questionnaire, which had to be filled out weekly, most participants adhered to this recommendation. The participants were considered adherent if they took at least ten capsules per week for three out of four weeks. Only three participants reported taking less than ten capsules per week on one of four intervention weeks. Therefore, all participants were considered adherent based on this definition.

Interestingly, the majority reported that they usually took both capsules simultaneously. The preferred time to take the capsules was in the morning. People who reported taking the two capsules at two different times during the day mostly took the capsules in the morning and in the evening.

### Fasting glucose level and HbA1c

**FIGURE 2:**
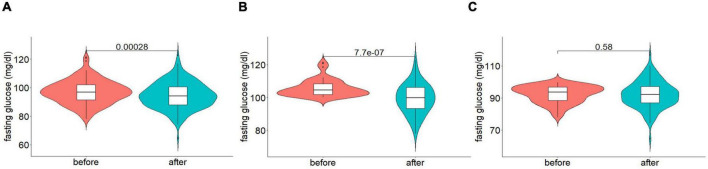
Fasting glucose before and after supplementation, **(A)** whole cohort, **(B)** prediabetic participants, and **(C)** participants without prediabetes. Data are presented as median ± IQR; p-values were estimated using Wilcoxon test.

To address whether the intervention influences glucose levels, fasting glucose levels were measured in the first and second sensor phases. Fasting glucose was determined as the baseline glucose level using a proprietary algorithm. Briefly, the baseline was predicted on daily individual 24-h glucose profiles and data from the medical anamnesis. The results showed a significant reduction in fasting glucose after intervention with the synbiotic-based supplement (*p* < 0.001). The fasting glucose level before the supplementation was 96.92 mg/dL (±8.29), whereas, after the supplementation, it decreased to 94.58 mg/dL (±9.27, Figure 2A and Table 2). As for HbA1c, the average was 5.72% (±0.27) before and decreased to 5.65% (±0.30) after the supplementation (*p* < 0.001, Table 3).

**TABLE 2:** Comparison of fasting glucose (mg/dL) before and after the intervention.

Participant	n	Before (mg/dL)	After (mg/dL)	*p*-value	Delta (%)
All	180	96.92 (±8.29)	94.58 (±9.27)	<0.001	–2.13 (±8.86)
Prediabetes	62	105.73 (±5.09)	99.23 (±9.12)	<0.001	–6.10 (±7.89)
No prediabetes	118	92.29 (±5.37)	92.14 (±8.39)	0.582	–0.05 (±8.65)

The delta (%) presented is a mean of the percentage change calculated for each participant. Data were analyzed with the Wilcoxon test.

**TABLE 3:** Comparison of HbA1c (in%) before and after the intervention.

Participant	n	Before (%)	After (%)	*p*-value	Delta (%)
All	180	5.72 (±0.27)	5.65 (±0.30)	<0.001	–1.20 (±4.72)
Prediabetes	62	6.01 (±0.19)	5.81 (±0.31)	<0.001	–3.31 (±4.36)
No prediabetes	118	5.58 (±0.17)	5.57 (±0.27)	0.449	–0.09 (±4.54)

HbA1c was calculated by multiplying the average glucose level of the complete test phase by 0.03 and adding 2.6 (32). The delta (%) presented is a mean of the percentage change calculated for each participant. Data ware was analyzed with the Wilcoxon test.

### Subgroup analysis of fasting glucose and HbA1c

The previous results raised a question whether fasting blood glucose was reduced in all participants or whether a specific group of participants carried the effect. Interestingly, in participants classified as prediabetic (*n* = 62) the fasting glucose level decreased from 105.73 mg/dL (±5.09) to 99.23 mg/dL (±9.12; *p* < 0.001, Figure 2B and Table 2) and Hb1Ac from 6.01% (±0.19) to 5.81% (±0.31; *p* < 0.001, Table 3). In 31 participants from the 62 that were classified as prediabetic before the trial, the fasting glucose value decreased to under 100 mg/dL (*p* < 0.001), and the HbA1c decreased under 5.7% (*p* < 0.001) after the trial. According to these two criteria, 50% (*n* = 31) of the prediabetic participants could no longer be classified as prediabetics at the end of the supplementation. In contrast, no significant change in fasting blood glucose (*p* = 0.582, Figure 2C and Table 2) or Hb1Ac (*p* = 0.449, Table 3) was observed in participants without prediabetes.

These results suggest that the possible effects of the synbiotic-based supplement on fasting blood glucose may depend on the baseline fasting blood glucose level. To investigate this hypothesis, a correlation analysis was performed. The analysis showed a significant correlation between the level of fasting blood glucose at baseline and the percentage change in fasting blood glucose (*R* = –0.38, *p* < 0.001). The higher the baseline value, the more significant was the decrease in fasting blood glucose post intervention.

### Postprandial glucose level

**FIGURE 3:**
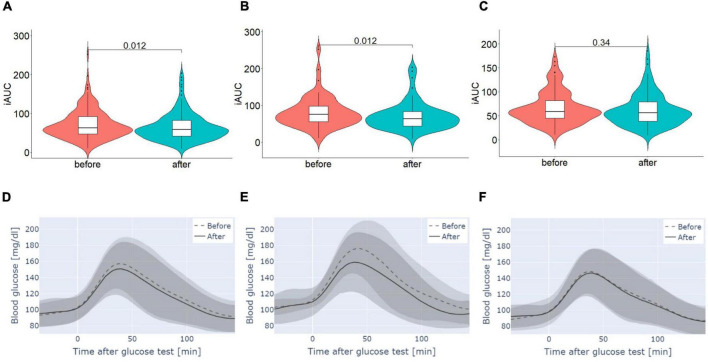
Glycemic responses to a standardized glucose test meal measured for 120 min determined by iAUC **(A–C)** and blood glucose level [mg/dL] **(D–F)** before and after intervention **(A,D)** whole cohort, **(B,E)** prediabetic participants and **(C,F)** participants without prediabetes. **(A–C)** Data are presented as median ± IQR, p-values were estimated using Wilcoxon test. **(D–F)** Data are presented as median and IQR.

To address the question of whether the daily intake of the supplement led to differences in postprandial glycemic responses, iAUC (incremental area under the curve) was determined for 120 min after the logged standardized glucose test meal intake. The comparison of glycemic response was restricted to participants who completed the glucose test meal in both sensor phases (*n* = 168). The results confirm a lower glycemic response after the supplementation (*p* = 0.012) (Figures 3A,D and Table 4).

**TABLE 4:** Comparison of responses to a standardized glucose test meal measured for 120 min determined by iAUC before and after the intervention.

Participant	n	Before	After	*p*-value	Delta (%)
All	168	71.44 (±37.39)	65.13 (±35.54)	0.012	–4.91 (±78.87)
Prediabetes	58	80.92 (±42.13)	71.37 (±38.60)	0.012	–6.28 (±115.85)
No prediabetes	110	66.44 (±33.78)	61.84 (±33.53)	0.345	–4.18 (±50.11)

The delta (%) presented is a mean of the percentage change calculated for each participant.

### Subgroup analysis of postprandial glucose level

The analysis of prediabetic participants showed a significant decrease in the glycemic response to the glucose test meal after supplementation with the investigational product (*p* = 0.012). In contrast, no significant change in glycemic response to a standardized glucose test meal of participants without prediabetes was found (*p* = 0.345). In addition, the changes (delta%) in glycemic responses over the course of the study were compared between participants with and without prediabetes. The percentage changes did not differ significantly between the two groups (*p* = 0.072, Figure 3 and Table 4).

### Comparison of body weight and composition

Participants were asked to report their current body weight and WHR at the beginning and end of the study. For this purpose, participants were provided with a measuring tape. Only participants whose fasting glucose was known and who reported their weight or WHR at both time points were included in this analysis (*n* = 171). Comparison of body weight at baseline and at the end of the study showed significant weight loss in participants. The average weight loss was 1.07 kg (±2.30; *p* < 0.001). Significant weight loss was also found in prediabetics and non-prediabetics. Here the differences before and after the study were 1.47 kg (±2.82; *p* < 0.001) and 0.87 kg (±1.97; *p* < 0.001) respectively. This weight loss was also reflected in a significant reduction in BMI, both in the total study population (*p* < 0.001) and in the groups of prediabetic (*p* < 0.001) and non-prediabetic participants (*p* < 0.001). In addition to body weight, it was also examined whether the WHR had changed. In contrast to body weight, no significant changes could be observed in the WHR. This was the case for the analysis of the study population as well as for the subgroups of prediabetics and non-prediabetics (Table 5).

**TABLE 5:** The absolute reduction of body weight, BMI and WHR throughout the study.

Variable	n	Before	After	*p*-value	Delta	Delta (%)
Body weight (kg)	171	80.71 (±18.00)	79.64 (±17.43)	<0.001^1^	–1.07 (±2.30)	–1.19 (±2.83)
Prediabetes	58	84.67 (±16.53)	83.21 (±15.94)	<0.001^1^	–1.47 (±2.82)	–1.61 (±3.21)
No prediabetes	113	78.68 (±18.44)	77.81 (±17.93)	<0.001^1^	–0.87 (±1.97)	–0.97 (±2.60)
BMI (kg/m^2^)	171	26.85 (±5.65)	26.50 (±5.47)	<0.001^1^	–0.35 (±0.78)	–1.16 (±2.84)
Prediabetes	58	27.28 (±4.86)	26.80 (±4.61)	<0.001^1^	–0.47 (±0.94)	–1.58 (±3.18)
No prediabetes	113	26.63 (±6.03)	26.35 (±5.88)	<0.001^1^	–0.28 (±0.68)	–0.94 (±2.64)
Waist-to-hip-ratio	171	0.87 (±0.10)	0.87 (±0.09)	0.621^2^	–0.002 (±0.05)	0.001 (±5.63)
Prediabetes	58	0.89 (±0.11)	0.89 (±0.10)	0.510^2^	–0.005 (±0.06)	–0.248 (±5.73)
No prediabetes	113	0.86 (±0.09)	0.86 (±0.09)	0.923^2^	–0.000 (±0.05)	0.129 (±5.60)

The delta presented is the absolute change calculated from mean before and after. The delta (%) presented is a mean of the percentage change calculated for each participant. Data were analyzed with ^1^paired *t*-test or ^2^Wilcoxon test.

### Influence of the supplement on the digestion of the participants

Participants completed a weekly abdominal well-being questionnaire that also contained the Bristol Stool Chart (27). The scores on the scale were compared at the beginning and end of the study to investigate whether regular intake the synbiotic-based supplement affected stool consistency and shape. The results of the analysis showed that the ratings on the scale at the end of the study did not differ significantly (*p* = 0.562). This indicates that stool consistency did not change over the course of the study. Stool frequency also remained relatively stable. In addition, the participants rated their feeling of hunger and satiety on a scale from 0 (very strong) to 10 (very weak). While the feeling of satiety remained unchanged, the feeling of hunger decreased significantly toward the end of the study (*p* < 0.001). Regarding digestive complaints, the descriptive results showed a slight increase in symptom-free subjects for all complaints except vomiting and reflux. Descriptively, the observed effect was most substantial for flatulence and abdominal pain.

### Microbiome analysis

A comparative analysis of the microbiome was performed during the first and last weeks of the intervention. Sequencing data (amplicon sequencing) from 180 volunteers were processed using dada2. Fourteen participants were excluded from the analysis as they only provided a stool sample from the one-time point. Two additional subjects were excluded because glucose data were not available. In total, data from 166 subjects (332 samples) were analyzed. Amplicon sequence variants (ASVs) that did not belong to bacteria or any known phylum were excluded, and ASVs belong to the phylum *Cyanobacteria*/*Chloroplast* or to the family *Mitochondria* were removed as well. After the pre-processing, on average 46.160 (±22.772) contigs were obtained, representing a taxonomic annotation.

**FIGURE 4:**
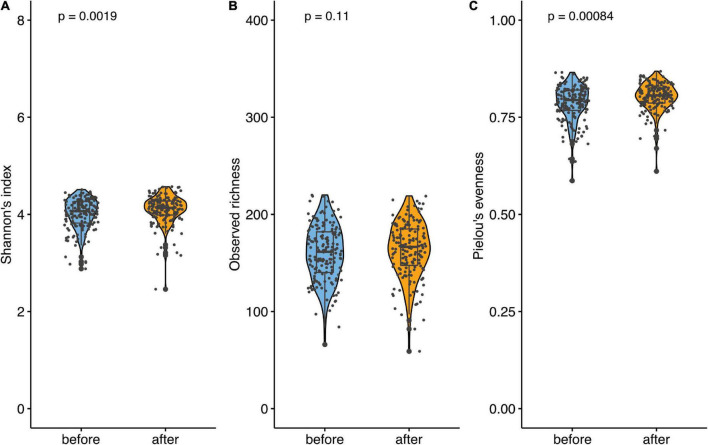
Microbiome analysis before and after the supplementation. **(A)** Comparison of alpha diversity. Alpha diversity was estimated using Shannon’s index. **(B)** Comparison of observed richness and **(C)** Pielou’s evenness. Data are presented as individual data points (dots); median and interquartile ranges are shown as boxplots; p-values were estimated using Wilcoxon test.

Alpha diversity was estimated using Shannon’s index and increased significantly over the course of the study (mean before = 4.012; mean after = 4.134; *p* = 0.002). This indicates a change in the number of species after the intake of the synbiotic-based supplement. Furthermore, observed richness and Pielou’s evenness were calculated based on rarefied data to avoid bias due to differences in sequencing depth. No significant differences in observed richness were found, but Pielou’s evenness shows a significant increase from before to after the intervention (Figure 4).

**FIGURE 5:**
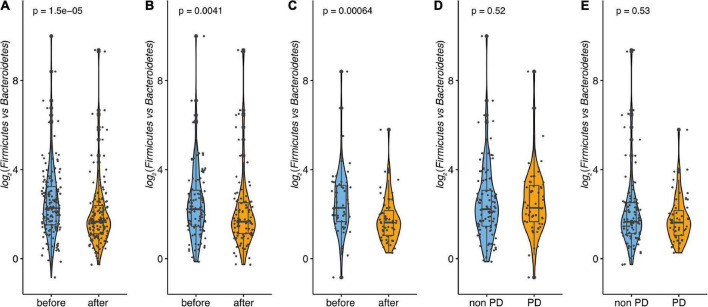
*Firmicutes*/*Bacteroidetes* (F/B) ratio. **(A)** Ratio F/B across all samples before and after. **(B)** Ratio F/B for non-pre-diabetic before and after. **(C)** Ratio F/B for pre-diabetic before and after. **(D)** Ratio F/B between non-pre-diabetic and pre-diabetic before. **(E)** Ratio F/B between non-pre-diabetic and pre-diabetic after. Data are presented as individual data points (dots); median and interquartile ranges are shown as boxplots; p-values were estimated using Wilcoxon test.

Beta diversity, estimated applying Aitchison distance, showed significant differences between the first and last week of the study (*p* = 0.010, R2 = 0.0039), indicating a substantial shift in the community. However, the effect was relatively small (0.39%). At the phylum level, we saw that *Bacteroidetes* increased over the course of the study (fdr = 0.007), while the abundance of *Firmicutes* decreased (fdr = 0.002). The ratio between *Firmicutes* to *Bacteroidetes* decreased significantly between the intervention’s start and end (Figure 5A).

### Subgroup analysis of microbiome

**FIGURE 6:**
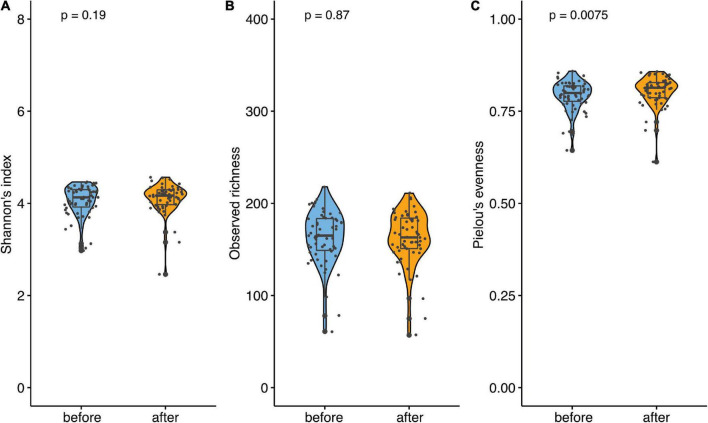
Microbiome analysis of prediabetic participants before and after supplementation. **(A)** Comparison of Alpha diversity. Alpha diversity was estimated using the Shannon index. **(B)** Comparison of observed richness and **(C)** Pielou’s evenness. Data are presented as individual data points (dots); median and interquartile ranges are shown as boxplots; p-values were estimated using Wilcoxon test.

For the microbiome, the subgroup analysis was performed as well. Similar to the microbiome data of the whole population, the alpha diversity, estimated using Shannon’s index, did not change over the course of the observation for the pre-diabetic subgroup (*p* = 0.190). The same applied to the observed richness which did not change between both measurements (*p* = 0.870). This indicates that the species number did not change significantly after the intake of the symbiotic-based product. However, Pielou’s evenness did differ significantly at the end of the study (*p* = 0.008, Figure 6). Interestingly, in the non-pre-diabetic population, alpha diversity, estimated using Shannon’s index, increased significantly over the course of the study (*p* < 0.001). This indicates a change in species number after the intake of the supplement. In line with the increase in the Shannon index, Pielou’s evenness was significantly higher at the end of participation (*p* < 0.001). Only the observed richness did not change (*p* = 0.21, Figure 6). Regarding beta diversity, estimated by Aitchison distance, no significant difference in community composition between the first and the last week of the study were found neither in pre-diabetics (*p* = 0.9997, R2 = 0.0057) nor in non-prediabetics (*p* = 0.7598, R2 = 0.00436). In the total population, we saw that *Bacteroidetes* significantly increased over the course of the observation and the abundance of *Firmicutes* decreased. This was also observed in pre-diabetic participants, on the phylum level, the average abundance of *Bacteroidetes* increased significantly, whereas the relative abundance of *Firmicutes* was considerably lower at the end of the observation. Interestingly, in non-pre-diabetes participants, no significant differences were seen in phylum levels between the start and end of the observation. Analyses of the ratio of *Firmicutes* to *Bacteroidetes* showed a significant decrease in non-prediabetic participants and prediabetic participants when compared from the start with the end of the observation (*p* < 0.01, Figures 5B,C). However, neither at the beginning nor the end of the study could the differences be observed compared to the prediabetics and non-prediabetic participants (Figures 5D,E).

## Discussion

An impaired glucose control manifested as elevated fasting plasma glucose level and/or impaired glucose tolerance is a silent but yet determining risk factor for developing T2D and cardiovascular diseases (CVD). The state of intermediate hyperglycemia, also known as prediabetes or non-diabetic hyperglycemia, describes the condition of impaired glucose tolerance and fasting glucose by elevated blood glucose levels above the normal range and below the diabetes diagnostic threshold (4). Early treatment may prevent or at least delay the onset of T2D in individuals with this condition (1–3). The clinically asymptomatic nature of this risk factor and the difficulty of adhering to lifestyle-based intervention strategies (28)-primarily targeting diet and physical activity-explain the continuing rise of T2D and CVD prevalence worldwide. These challenge societies in terms of economic damage from increased morbidity and health care costs. It clearly shows the need for developing novel strategies that fulfill the criteria of effectiveness, convenience, safety, and affordability.

We started to conceive such a strategy in form of a dietary supplement based on the synbiotic formulation comprising *Bacillus subtilis* DSM 32315 and L-alanyl-L-glutamine, as reported previously (21). In a pilot study, this synbiotic was shown to increase butyrate levels *in vitro* as well as *in vivo*, accompanied by glucose-, cholesterol-, and satiety hormone-modulating effects (21). While the pilot study population involved only men (*n* = 18) of younger age, normal weight, and regular fasting blood glucose on average (21), the cohort of the present study was more representative of the general population in terms of gender, age, weight, and glucose control. This cohort, though generally healthy, had a considerable percentage of participants classified as overweight or obese (51.57%, *n* = 99), and prediabetic (32.29%, *n* = 62), and was therefore considered suitable to replicate the anti-hyperglycemic and putative weight-modulating effects of the synbiotic. The pre-diabetic participants in our study were defined by IFG concentrations of 100-125 mg/dL and a HbA1c level of 5.7% to 6.4%. The IFG value defining prediabetes was adjusted from 110 to 100 mg/dL in 2003 by ADA and is still subject to controversies (29). The ADA argues that the adjusted value is less sensitive but more specific and thus has a higher positive predictive value to identify people at risk for later development of T2D (4). Our data demonstrate that the intake of the novel study product had a relevant impact on improving risk factors for metabolic disorders in a general German population cohort. Four weeks of daily supplement intake resulted in reductions in fasting blood glucose, calculated HbA1c levels, body weight, and improvements in glucose tolerance. Notably, a subgroup analysis revealed that participants defined as pre-diabetics at baseline benefited the most. The significant changes in these parameters referred to this subgroup (*n* = 62, 34.4% of the total cohort), while the healthy participants remained within their initial normoglycemic range, which indicates the safety of the study product for both subgroups.

The idea of using probiotics for glucose control is not new. In the past, specific attention has been paid to butyrate-producing bacteria such as *Faecalibacterium prausnitzii*, *Oscillibacter*, and *Clostridium XIVa*. These bacteria use mainly pyruvate/acetyl CoA pathway (Ac pathway) which leads to butyrate as the end product. The short-chain fatty acid butyrate is the most widely studied microbial metabolite in regard to glucose homeostasis and metabolism. In the gut, butyrate acts as a ligand for metabolite-sensing G-protein coupled receptors (GCPRs), including GPR43, GPR41, and GPR109 which stimulates glucagon-like peptide 1 (GLP-1) and peptide YY (PYY) (29). While GLP-1 mediates an increase in insulin secretion and regulates blood glucose concentrations, PYY reduces appetite (30, 31). Moreover, islets are known to express the butyrate receptors GPR41 and 43, indicating that butyrate might be involved in islet-cell metabolism and function (32). In the before mentioned pilot study with the same synbiotic-based supplement, an increase of 21% in butyrate levels in stool samples was observed, which could be attributed to the expansion of few butyrate-producing taxa, including *Faecalibacterium prausnitzii*. In contrast, in the present study, the glucose control changes could not be linked to shifts in participants’ microbiome compositions. This might be explained by great interindividual variability in gut microbiota and host responsiveness, thus complicating the prediction of gut microbiota and host response to a given dietary intervention (33). The only microbiome effect in the current investigation was a shallow change in the *Firmicutes*/*Bacteroidetes* ratio when comparing the start and the end of the observation in prediabetic participants. We conclude that interindividual variability in gut microbiota composition and responsiveness as well as technical confounders make it challenging to predict their modulation in response to a given dietary intervention (33) based on group analyses.

Probably, since prediabetes can in principle be reverted by life-style changes, studies focusing on the treatment of this condition remain scarce. Speaker et al. showed that between 2011 and 2018 only 20% of patients diagnosed with prediabetes obtained a treatment. If treated, the medical nutrition therapy or metformin were prescribed (34). To our knowledge, the study presented here is the first to show a positive effect of a probiotic (or synbiotic)-based supplement in this population. However, probiotics have been investigated in patients with T2D. For instance, Perraudeau et al. investigated the effect of the probiotic formulation (*A. muciniphila, C. beijerinckii, C. butyricum, B. infantis* and *A. halli*) on the glycemic control in T2D subjects with metformin monotherapy (35). This 12-week randomized clinical trial (RCT) resulted in reduction of fasting glucose by 3 mg/dL, postprandial glucose by 11.7 mg/dL/180 min and HbA1c by 0.2% in participants taking the probiotic. Intake of the synbiotic investigated in the present study for four weeks resulted in a greater fasting glucose reduction (by 6.50 mg/dL), however, the postprandial levels decreased to a lower extent (by 9.55 mg/dL/120 min). The Hb1Ac decreased in our study by 0.20% as well; however, it has to be noted that as HbA1c measures an approximate 3-month average glucose concentration in blood, it is challenging to draw a conclusion regarding the extent of the change in a study of only 6 weeks duration. Interestingly, similarly to our study Perraudeau et al. could not attribute changes in the glucose metabolism to changes in microbiome even though the slight increase in butyrate levels was detected. In contrast to this RTC study, our study was performed in a non-clinical, real-life setting under habitual living conditions and application of a CGM device, similarly to a recently conducted study (36). No additional invasive or interventional procedure were performed, so the butyrate levels and additional blood parameters were not measured. This approach was chosen to investigate potential efficacy even under these less controlled everyday conditions assuming a good transferability to real-life afterward, which might add additional insights besides the results of RCTs. While they are the gold standard for evidence-based medicine, they do not always reflect real-world populations (in particular with respect to compliance), limiting their generalizability and external validity (37, 38). This setting provided data from a relatively large population cohort, which integrated the study product into their everyday lifestyle and diet. Further, as blood glucose regulation is highly variable and subject to interindividual confounders (13, 39), CGM is advantageous over single time point assessments of blood glucose levels.

Our investigation has some limitations. Due to its non-clinical design with only one study arm, placebo effects cannot be excluded. Standard techniques did not validate blood glucose data collected by the CGM device, and other related parameters (e.g., blood lipids, satiety hormones, fecal butyrate) were unavailable because only a retrospective analysis of this non-invasive investigation was conducted. The proposed mode-of-action via increased *in vivo* butyrate production by a modulation of intestinal microbiota composition can only be hypothesized based on former findings (20). As no other invasive blood drawings were included in the study, HbA1c values were not determined by laboratory analyses but were calculated based on the CGM data. HbA1c, as calculated in this study, is therefore not of the same quality as HbA1c measured in standardized laboratories. The study product –a dietary supplement marketed to healthy consumers- contained additional ingredients (vitamins, zinc, curcumin, and green tea extracts). Therefore, the effects observed here cannot solely be assigned to the synbiotic combination.

In summary, this retrospective data analysis shows that the synbiotic-based product effectively manages glucose levels in prediabetic populations in a real-life setup. However, additional placebo-controlled and appropriately powered studies comparing the synbiotic formulation with and without the other ingredients are necessary to elucidate the active ingredients and to reveal the mode(s) of action(s).

## Conclusion

With this retrospective data analysis from a real-life setting, we reinforced our initial findings of metabolic effects elicited by a novel synbiotic formulation comprising *Bacillus subtilis* DSM 32315 and L-alanyl-L-glutamine. More specifically, we showed that the formulation improves the postprandial glucose response, fasting glucose level, calculated HbA1c, and body weight, especially in individuals at risk of developing diabetes or metabolic syndrome. The effects observed were robust even in such a very heterogeneous population and not overruled by the habitual diet and usual lifestyle. In conclusion, this study supports that the product may be a successful strategy for hyperglycemia and metabolic syndrome (MetS) management. It is worthwhile to investigate the synbiotic further in subjects with MetS or T2D, also in combination with standard therapeutic strategies in additional trials.

## Data availability statement

The data presented in this study are deposited in the European Nucleotide Archive repository, accession number PRJEB55518.

## Ethics statement

Ethical review and approval was not required for the study on human participants in accordance with the local legislation and institutional requirements as it was post-marketing observation. The Ethical Committee of the University of Lübeck approved the retrospective analyses of the data. The patients/participants provided their written informed consent to participate in this study.

## Author contributions

HD contributed to conception and design of the study. FS and TS did methodology. AKü, FS, AKo, and VT-L did formal analysis. AKo, VT-L, HD, and BS wrote and prepared the first draft of the manuscript. CS, MS, AKü, GL, and TS reviewed and edited the manuscript. All authors contributed to manuscript revision, read, and approved the submitted version.

## Footnotes

1
www.millionfriends.de
