# Supplementary figures and images for: A nutritional supplement based on a synbiotic combination of Bacillus subtilis DSM 32315 and L-alanyl-L-glutamine improves glucose metabolism in healthy prediabetic subjects – A real-life post-marketing study
Source: Front Nutr. 2022 Dec 8;9:1001419. doi: 10.3389/fnut.2022.1001419 (PMC9773202; doi:10.3389/fnut.2022.1001419)

Table S2: Demographics of prediabetic participants (n=62). Distribution of gender, age and weight

 
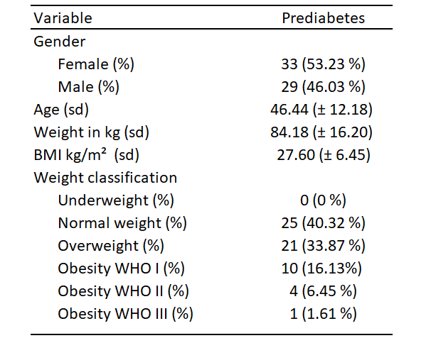

Supplement: Supplementary file 2 [file Table_2.docx]
