# Supplementary material for: A nutritional supplement based on a synbiotic combination of Bacillus subtilis DSM 32315 and L-alanyl-L-glutamine improves glucose metabolism in healthy prediabetic subjects – A real-life post-marketing study
Source: Front Nutr. 2022 Dec 8;9:1001419. doi: 10.3389/fnut.2022.1001419 (PMC9773202; doi:10.3389/fnut.2022.1001419)

Table S3: Mean values of macronutrient intake, average glucose values, and physical activity for participants with prediabetes (n=62) in both sensor-assisted test phases (first sensor phase = before; second sensor phase = after).


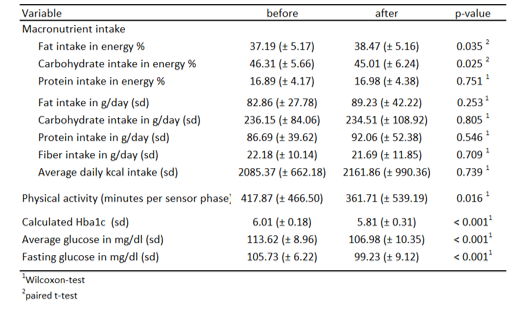

Supplement: Supplementary file 3 [file Table_3.docx]
